# Effect of Prior Direction Expectation on the Accuracy and Precision of Smooth Pursuit Eye Movements

**DOI:** 10.3389/fnsys.2019.00071

**Published:** 2019-11-26

**Authors:** Seolmin Kim, Jeongjun Park, Joonyeol Lee

**Affiliations:** ^1^Center for Neuroscience Imaging Research, Institute for Basic Science, Suwon, South Korea; ^2^Department of Biomedical Engineering, Sungkyunkwan University, Suwon, South Korea

**Keywords:** Bayesian inference, smooth pursuit, macaque, likelihood function, prior probability

## Abstract

The integration of sensory with top–down cognitive signals for generating appropriate sensory–motor behaviors is an important issue in understanding the brain’s information processes. Recent studies have demonstrated that the interplay between sensory and high-level signals in oculomotor behavior could be explained by Bayesian inference. Specifically, prior knowledge for motion speed introduces a bias in the speed of smooth pursuit eye movements. The other important prediction of Bayesian inference is variability reduction by prior expectation; however, there is insufficient evidence in oculomotor behaviors to support this prediction. In the present study, we trained monkeys to switch the prior expectation about motion direction and independently controlled the strength of the motion stimulus. Under identical sensory stimulus conditions, we tested if prior knowledge about the motion direction reduced the variability of open-loop smooth pursuit eye movements. We observed a significant reduction when the prior expectation was strong; this was consistent with the prediction of Bayesian inference. Taking advantage of the open-loop smooth pursuit, we investigated the temporal dynamics of the effect of the prior to the pursuit direction bias and variability. This analysis demonstrated that the strength of the sensory evidence depended not only on the strength of the sensory stimulus but also on the time required for the pursuit system to form a neural sensory representation. Finally, we demonstrated that the variability and directional bias change by prior knowledge were quantitatively explained by the Bayesian observer model.

## Introduction

When we interact with a dynamic environment, we combine multiple pieces of available information to make proper behavioral responses. Each piece of information is weighted by its reliability when it is integrated with others. In what follows, we will refer to this reliability as precision. Precision is the inverse variability or dispersion of a probability distribution, such that a narrow distribution is precise. For example, when we drive a car on a clear day, we primarily rely on visual information because the visual sensory evidence is precise. However, on a rainy day or at night, our dependence on other information, such as prior knowledge about the road, will increase because the incoming sensory evidence is imprecise. The Bayesian inference framework provides a quantitative description of this reliability-based integration. Using Bayes’ rule, a simple multiplication of probability distributions automatically provides the optimal integration as well as several predictions. First, our behavior will be biased toward the prior expectation of the sensory stimulus when the sensory evidence is imprecise. Second, for identical imprecise sensory information, the reliability of the behavior will be higher when the prior expectation is strong.

Bayesian inference has been successful in explaining not only perceptual and cognitive processes (Kording and Wolpert, 2006; Stocker and Simoncelli, 2006; Berniker and Kording, 2011; Ma, 2012) but also several interesting properties of simple sensory–motor behaviors (Montagnini et al., 2007; Freeman et al., 2010; Bogadhi et al., 2011, 2013; Verstynen and Sabes, 2011; Simoncini et al., 2012; Yang et al., 2012; Orban de Xivry et al., 2013; Darlington et al., 2017, 2018; Deravet et al., 2018). The interplay between prior knowledge and sensory evidence was demonstrated in smooth pursuit eye movements by a neurally plausible network model (Yang et al., 2012). More recent studies demonstrated that the integration of a short-term prior for speed and sensory information in smooth pursuit can be explained by Bayesian integration in monkeys (Darlington et al., 2017, 2018) and humans (Deravet et al., 2018). These studies primarily investigated the interaction between prior knowledge for motion speed and the precision of the visual motion, and they demonstrated the attraction of pursuit speed toward the prior information (bias). However, few studies have investigated the effect of prior expectation on the variability of oculomotor behavior, which is the other important prediction of Bayesian inference. An exception here is the work of Adams et al. (2015, 2016) who used an active inference to model pursuit eye movements of a sinusoidal target (with occlusion). By fitting the scheme to eye-tracking data, they were able to infer the precision afforded sensory information and prior expectations. Furthermore, using magnetoencephalography during the same paradigm, the authors were able to assign gain control at different levels in the visual hierarchy to subjective precision under ideal Bayesian observer assumptions.

In the present study, we tested if the prior expectation about motion direction contributed to more precise smooth pursuit eye movements. We developed an experimental design for independent control of the strength of prior expectation about motion and the precision of sensory information, and we trained monkeys on a task in which they switch their prior expectations in different trial blocks. By evaluating the effect of the switching prior for identical sensory stimulus, we were able to evaluate whether strengthening the prior expectation indeed decreases the variation in pursuit direction, as the Bayesian inference model predicted. We observed that the variability of open-loop smooth pursuit was significantly reduced when the prior expectation about motion direction was strong, and this reduction was larger when the sensory motion was imprecise, along with the attraction of pursuit traces toward the prior direction. Furthermore, we showed that the strength of the sensory evidence depended not only on the precision of visual motion, but also on the time required for the system to form a motion representation – i.e., accumulate evidence for posterior beliefs – in the brain. Finally, the Bayesian observer model explained the interaction between the prior expectation and sensory evidence. This suggests that the initiation of smooth pursuit can be explained by Bayesian inference.

## Materials and Methods

Two male rhesus monkeys (*Macaca mulatta*) that weighed between 9.5 and 10.5 kg were trained on a smooth pursuit eye movement task. Before the training, we performed surgery to implant a head holder on the skull for head restraint. The surgery was carried out under aseptic conditions using isoflurane anesthesia and was followed by administration of antibiotics and analgesics to minimize postsurgical infection and pain. All research protocols were approved by the Sungkyunkwan University Institutional Animal Care and Use Committee.

### Data Acquisition

We presented visual stimuli on a gamma-corrected 24-inch CRT monitor (HP1230, 1600 × 1200 pixels, 85 Hz vertical refresh rate). The monitor was placed 570 mm from the animal, and it covered a 38.67°× 29.49° (horizontal × vertical) visual field. All stimuli were presented on a gray background (38.48 cd/m^2^) on a gray scale that covered a luminance range from 0 cd/m^2^ to 73.68 cd/m^2^. The horizontal and vertical components of the eye position were recorded at a sampling rate of 1000 Hz using an infrared video tracking system (EyeLink 1000 plus, SR Research Ltd). The visual stimulus presentation and data acquisition were controlled by a real-time data acquisition system (Maestro version 3.3.1^1^). To obtain the timing of the visual stimuli accurately, we used a custom-built photodiode system.

### Task Design and Visual Stimuli

**FIGURE 1:**
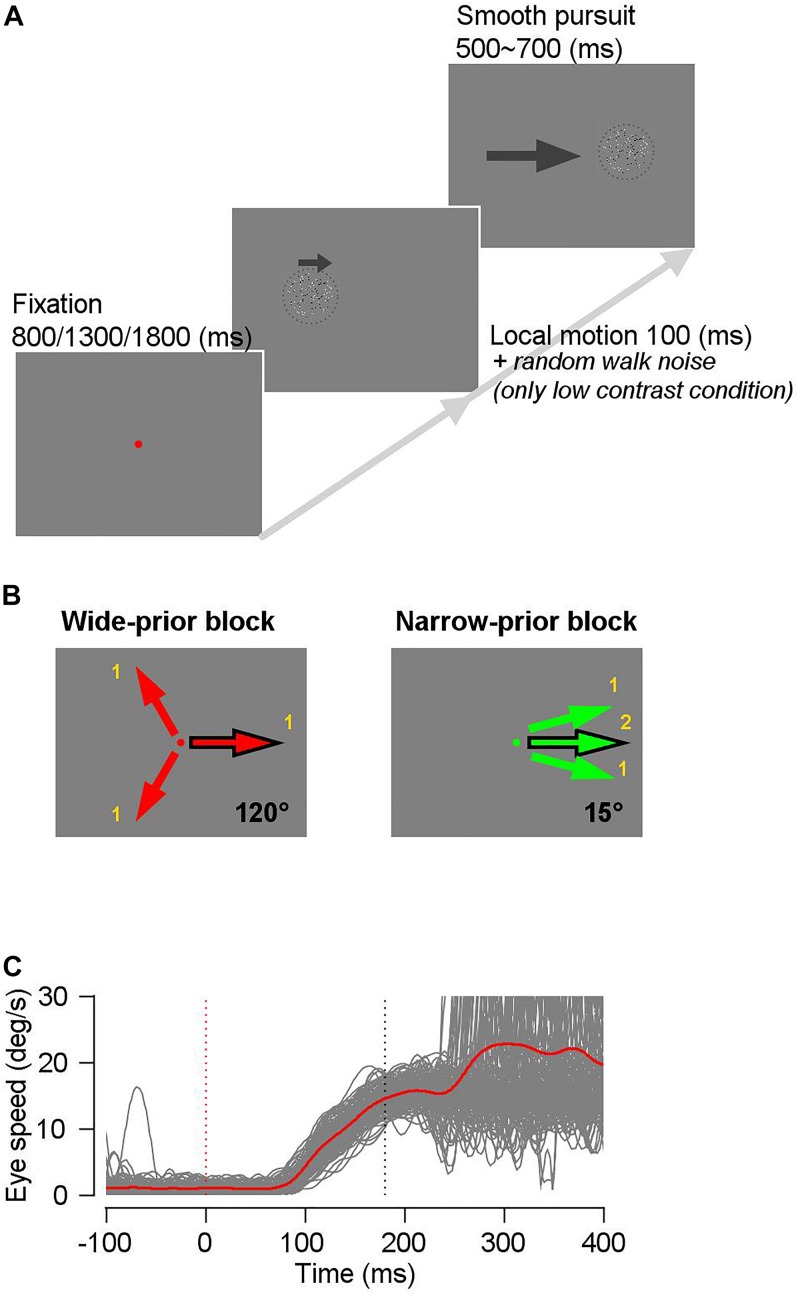
Schematics of the task. **(A)** A trial started with onset of a red or green fixation spot. The fixation duration was randomly selected from three time periods (800/1300/1800 ms) to prevent monkeys from anticipating the stimulus onset timing. A random-dot kinematogram appeared after the extinction of the fixation spot, and for 100 ms, only dots inside the invisible circular window moved into one of three predetermined directions, whereas the invisible window did not move. Then, the dots and the window moved together for 500–700 ms. **(B)** In a wide-prior block, the motion direction in each trial was one of three evenly spaced directions (120**°** apart from one another), whereas in a narrow-prior block, the motion direction in each trial was one of three narrowly spaced directions (15**°** apart from one another). The common motion direction between the two blocks is denoted by a black outlined arrow. **(C)** Example eye speed traces after removing trials with saccadic eye movements in a time window between 0 ms and 250 ms from stimulus onset. Gray traces show individual trials and a red trace shows the average eye speed.

We trained the two monkeys on a modified version of the step-ramp pursuit task (Rashbass, 1961). Figure 1 shows the task design. We used a random dot kinematogram as a pursuit target; the dot patch consisted of an equal number of bright and dark spots inside an invisible circular window. Therefore, the average luminance of the dot patch was always the same as that of the gray background. The dot density was 5 dots/deg^2^. The nominal contrast of the dot patch was defined as the difference between the luminance of the bright and dark dots, divided by the sum of their luminance. We controlled the animal’s prior expectation about the motion direction by blocks of trials in which we changed the configuration of motion directions (see below). We also controlled the luminance contrast and/or the directional “random walk” noise (Osborne and Lisberger, 2009) of the pursuit target to change the precision of the sensory evidence. Each trial started with the onset of a fixation spot. In one block (narrow-prior block), we used a green fixation spot, and in the other block (wide-prior block), we used a red fixation spot so that animals could know in which block they were. After the monkeys fixated on the spot within 1.25° and randomly selected one of three fixation durations (800, 1300, and 1800 ms), the random dot kinematogram appeared at the center of the screen or was displaced by approximately 1 ∼ 2° in the direction opposite of that of the on-going pursuit. For the first 100 ms, all the dots inside the invisible static circular aperture (4° by 4° or 6° by 6°) moved at a given speed (10°/s ∼ 20°/s) and in one of the predetermined directions (a local motion). Subsequently, all the dots and the aperture moved in the same direction and at the same speed with the local motion. In the narrow-prior block, the direction of the pursuit target in each trial was one of the three narrowly distributed directions (a prior direction and the prior direction ± 15°), as seen in Figure 1B. In this block, we presented the prior direction twice as often as the other directions to expedite the development of a direction prior. The movement of the dots and the aperture lasted for 500 ∼ 700 ms, and if the animals maintained their eyes within a 4°-window from the center of the patch until the end of each trial, then they were rewarded by drops of juice or water. In the wide-prior block, the pursuit targets were identical to those in the narrow-prior block, except that the direction of the pursuit target in each trial was selected from three directions that were 120° apart from one another (Figure 1B). We always included a direction that is the same as the prior direction so that we can compare the effect of the direction prior to the behavior under identical sensory stimulus conditions (Figures 1A,B). For example, if the direction that was common across the blocks was 0°, then the wide-prior block involved the directions 120°, −120°, and 0°, and the narrow-prior block involved the directions 15°, −15°, and 0°. In both blocks of trials, we randomly interleaved two types of pursuit targets. One was always with 100% contrast (high-contrast target) and the other was with 8%, 10%, or 12% contrast (low-contrast target). To reduce the precision of the motion signal under low-contrast stimulus further, we included a random-walk direction noise (±60°) in the low contrast patch in some days’ experiments (Osborne and Lisberger, 2009). In monkey A, one of the three low-contrast stimuli (8%, 10%, or 12%) were used in a given day’s experiment, and in monkey B, we used only 8% luminance contrast stimulus as the low-contrast target. For low-contrast stimuli, the results were qualitatively the same across the specific stimulus conditions. Therefore, we did not specify the exact stimulus conditions but rather termed them as “high contrast” and “low contrast.” The coherence of the pursuit targets was 100%, and the lifetime of the dots was infinite. The narrow-prior block consisted of 252 trials and the wide-prior block consisted of 378 trials. In each day’s experiment, we alternated the two blocks, starting from either the narrow-prior or wide-prior block. We typically collected four blocks for each category, resulting in 2520 trials in total.

### Data Analysis

To quantify smooth pursuit eye movements, we obtained the eye velocity by differentiating the horizontal and vertical eye position data recorded from the infrared video tracking system. Before the differentiation, we excluded any high-frequency components by applying a second-order low-pass Butterworth filter with a cut-off frequency of 20 Hz using a subroutine of the FieldTrip Matlab package. Then, we excluded trials with saccadic eye movements in a time window between −100 ms and 250 ms from target onset [Figure 1C, open-loop period (Lisberger et al., 1987)]. Subsequently, following Lee and Lisberger, 2013, we decomposed the eye velocity during the “open-loop” period into direction, speed, and latency components. That is, we obtained a template by averaging the horizontal and vertical velocity components of a given pursuit direction between −20 ms and 100 ms from the average pursuit latency and rotating the trials so that the average pursuit direction would be 45°. As was previously explained, we rotated the eye traces to obtain an unbiased measure of the pursuit direction in each trial. Then, we estimated the best-fitted latency and scaling factors of the horizontal and vertical eye velocity components in each trial by sliding and scaling the two templates simultaneously. The least-squares method and the NOMAD algorithm (Le Digabel, 2011) were used in the estimation. We included trials for further analysis only if the fitted function accounted for more than 70% of the data variance. After this procedure was carried out, we used the results for subsequent analysis only if the number of remaining trials of the prior direction in each block for the day’s experiment was more than 70.

### Bayesian Observer Model

Bayes’ rule states that the posterior probability distribution is proportional to the product of the likelihood function and the prior probability distribution:

(1) $$\text{p}\left(\theta_{s}|\theta_{m}\right)\propto \text{p}\,\left(\theta_{m}|\theta_{s}\right)\times p\,\left(\theta_{s}\right)$$

where p(θ_*s*_|θ_*m*_) is the posterior probability distribution for the target direction, θ_*s*_ is the stimulus direction, θ_*m*_ is the observed stimulus, p(θ_*m*_|θ_*s*_) is the likelihood function, and *p*(θ_*s*_) is the prior probability distribution for the target direction. In this study, we assume Gaussian distributions for the prior probability and likelihood function, and therefore the posterior probability distribution also follows a Gaussian distribution, that is:

(2) $$p(\theta s|{\text{θ}}_{m})\propto \frac{1}{\sqrt{2\text{π}}{\text{σ}}_{m}}e^{-\frac{{\left({\text{θ}}_{s}-{\text{θ}}_{m}\right)}^{2}}{2{\text{σ}}_{m}^{2}}}\frac{1}{\sqrt{2\text{π}}{\text{σ}}_{p}}e^{-\frac{{\left({\text{θ}}_{s}-{\text{θ}}_{p}\right)}^{2}}{2{\text{σ}}_{p}^{2}}}=\frac{1}{{\text{2πσ}}_{m}{\text{σ}}_{p}}e^{-\frac{{\text{σ}}_{p}^{2}{\left({\text{θ}}_{s}-{\text{θ}}_{m}\right)}^{2}+{\text{σ}}_{m}^{2}{\left({\text{θ}}_{s}-{\text{θ}}_{p}\right)}^{2}}{2{\text{σ}}_{m}^{2}{\text{σ}}_{p}^{2}}}$$

where σ_*m*_ is the standard deviation (SD) of the likelihood function, σ_*p*_ is the SD of the prior probability distribution, and θ_*p*_ is the mean of the prior probability distribution. The maximum *a posteriori* (MAP) estimate from this posterior probability distribution of Gaussian shape will be:

(3) $$\theta_{e\,s\,t}=\frac{\sigma_{p}^{2}}{\sigma_{p}^{2}+\sigma_{m}^{2}}\,\theta_{m}+\frac{\theta_{p}\,\sigma_{m}^{2}}{\sigma_{p}^{2}+\sigma_{m}^{2}}$$

Note that the weightings of the measured variable and prior variable depend upon their relative variances (i.e., standard deviations squared). Equivalency, the relative weighting of sensory evidence and prior expectations depends upon their precision (i.e., inverse variance). This is a generic feature of Bayesian filtering or evidence accumulation (Ernst and Banks, 2002); namely, the gain or precision afforded sensory evidence has a profound effect on posterior beliefs. We will see this below in terms of the standard deviation of the most likely posterior estimates.

The MAP estimate of the target direction will be a function of θ_*m*_, and θ_*m*_ itself is a random variable whose mean is equal to the stimulus direction, and its SD is equal to σ_*m*_. Therefore, the mean and SD of the MAP estimate can be calculated using the following equations:

(4) $$\text{E}\,\left(\theta_{e\,s\,t}\right)=\frac{\sigma_{p}^{2}}{\sigma_{p}^{2}+\sigma_{m}^{2}}\,\bar{\theta_{m}}+\frac{\theta_{p}\,\sigma_{m}^{2}}{\sigma_{p}^{2}+\sigma_{m}^{2}}$$

(5) $$\text{STD}\,\left(\theta_{e\,s\,t}\right)=\left(\frac{\sigma_{p}^{2}}{\sigma_{p}^{2}+\sigma_{m}^{2}}\right)\,\sigma_{m}$$

Then, the mean and SD of the MAP estimates were compared with those of the open-loop pursuit directions. We assumed that sensory representations for different motion directions would be different; therefore, we used different values of σ_*m*_ for each motion direction. To model the change in the likelihood function as the contrast/stimulus pattern changes, we used a multiplicative scaling factor that was attached to σ_*m*_. Therefore, we had eight free parameters: five σ_*m*_s for the motion directions, one scaling factor for contrast/stimulus, σ_*p*_ for the prior probability distribution in the narrow-prior block, and another σ_*p*_ for the prior probability distribution in the wide-prior block. We used 24 data points for parameter estimation: 12 means and 12 SDs of pursuit direction (three directions × two contrasts/stimulus patterns × 2 prior conditions). We estimated the model parameters using the “fmincon” function of MATLAB (Mathworks, Natick), where the algorithm determines the parameters that minimize the sum of squared errors (a least-squares method).

## Results

Our goal in this study was to test if monkey’s prior expectation adjusts the accuracy and precision of sensory-motor behavior consistent with the prediction of the Bayesian observer model. We trained two rhesus monkeys on a smooth pursuit eye movement task in which we independently controlled the strength of visual motion and each monkey’s prior expectation about the motion direction. We used a block design to control the prior expectation of the monkeys (narrow-prior block vs. wide-prior block, see section “Materials and Methods” for details). Using this design, we evaluated the effect of the direction prior on the bias and variability of the pursuit direction, the temporal dynamics of the interaction between prior and sensory evidence, and the quantitative correspondence with the Bayesian observer model.

### Effect of Prior Expectation on the Bias and Precision of Smooth Pursuit Direction Traces

**FIGURE 2:**
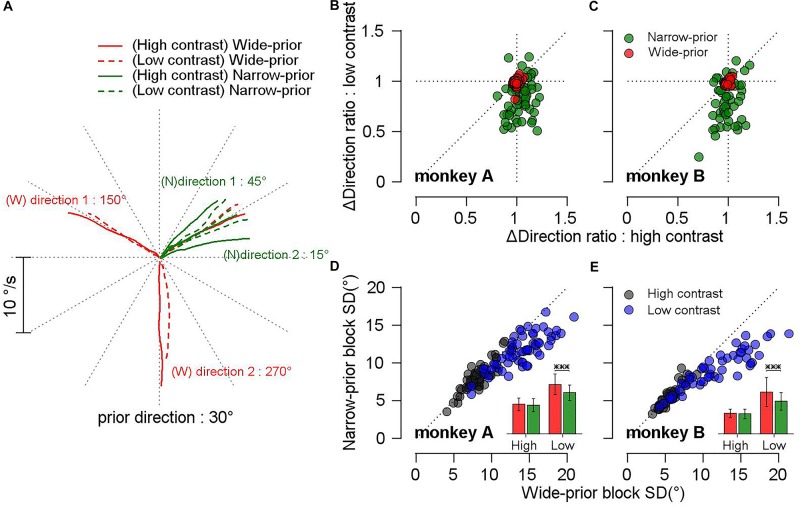
Effect of prior expectation and strength of sensory evidence on the precision and bias of directions of smooth pursuit eye movements. **(A)** Example average eye velocity traces from a day’s experiment plotted in Cartesian coordinate. In this example, the common prior direction was 30°. Red colors show the traces in the wide-prior block, and green colors show the traces in the narrow-prior block. Solid lines show the traces for 100% contrast target stimulus and dotted lines showed the traces for 8% contrast target stimulus. **(B,C)** Scatter plots comparing the ratios of direction differences between the two blocks. The *x*-axis denotes the ratios under high-contrast target motion, and the *y*-axis denotes the ratios under low-contrast target motion. Red filled circles show the ratios in the wide-prior block, and green filled circles show the ratios in the narrow-prior block. **(D,E)** Scatter plots showing the SD of pursuit directions for the common prior direction. The *x*-axis shows the SD in the wide-prior block, and the *y*-axis shows the SD in the narrow-prior block. Gray filled circles are data corresponding to high-contrast motion stimulus, and blue filled circles are data corresponding to low-contrast motion stimulus. Insets show a comparison of the SD between the two blocks. Red bars show the SD in the wide-prior block, and green bars show the SD in the narrow-prior block. Error bars denote the SD across experimental sessions. ^∗∗∗^*p* < 0.001.

A previous study (Yang et al., 2012) reported that monkeys can develop a prior expectation about the stimulus direction after a week of training. In our study, it was demonstrated that monkeys could switch their prior expectations across the blocks of trials in a single day. In the narrow-prior block, the pursuit velocity traces of both monkeys were attracted toward the central prior direction, especially when the luminance contrast of the stimulus was low, whereas no strong attractions were observed in the wide-prior block. Figure 2A shows the mean eye velocity traces of monkey A in a given day’s experiment. In this example, the motion direction that was identical between the two blocks was 30°. When the contrast of the pursuit targets was high, the monkey’s eye traces matched well with the target velocity traces (solid lines). However, when the contrast was low, the pursuit velocity traces for the two outer directions (15° and 45°) in the narrow-prior block (green dotted lines) were attracted toward the central direction (30° in this case). In the wide-prior block, the pursuit velocity traces for the two outer directions (150° and 270°) in low and high contrast matched well with the target directions although there was a slight tendency toward 30° when the pursuit target was at 270° and the luminance contrast was low. To summarize the effect of prior expectation on the pursuit directions across multiple days’ data in the two monkeys, we calculated the distance between two outer target directions and the distance between the corresponding pursuit directions. Then, we quantified the effect of prior expectation by the ratio between the two distances. If the pursuit traces were attracted toward the central direction, the ratio would be smaller than 1. In the narrow-prior block (Figures 2B,C, green color), the ratios of both monkeys were significantly smaller than 1 when the stimulus contrast was low (mean ratio 0.83 and 0.79, and *t*-test on log-transformed data, *p* = 3.46 × 10^–10^ and 3.84 × 10^–8^ for monkeys A and B, respectively). When the stimulus contrast was high, the effect of prior expectation had a repulsive effect (monkey A, mean ratio = 1.04, *t*-test on log-transformed data, *p* = 0.004), or the effect was not significant (monkey B, mean ratio = 0.996, *t*-test on log-transformed data, *p* = 0.54). In the wide-prior block, the ratios were centered at 1 regardless of the stimulus contrast in both animals (Figures 2B,C, red color) although the prior direction expectation had an effect on the mean of the pursuit direction traces. When the stimulus contrast was low, the effect of the prior expectation was significant in both animals (mean ratio 0.987 and 0.991, and *t*-test on log-transformed data, *p* = 0.004 and 0.01 for monkeys A and B, respectively). When the stimulus contrast was high, the effect was not significant (mean ratio 1 and 0.995, and *t*-test on log-transformed data, *p* = 0.67 and 0.1 for monkeys A and B, respectively). These results are consistent with the prediction of Bayesian inference. When sensory evidence is precise, prior expectation about direction does not have strong influence on the behavior. However, when sensory evidence is imprecise, prior expectation has an effect on the behavior by increasing bias toward the prior direction.

The other important feature of Bayesian inference is the change in the reliability of behavior. In the perception task, it is difficult to have direct access on the reliability of behavior; therefore, this is usually inferred from, for example, the psychometric curve. In smooth pursuit eye movements, we can evaluate the trial-by-trial variability of the underlying processes by directly observing the direction variation of smooth pursuit initiation. This aspect of Bayesian inference was investigated in a previous study (Yang et al., 2012) but was interpreted as a result of “signal-dependent” noise (Harris and Wolpert, 1998). In that study, only the relationship between pursuit bias and variance across multiple days and stimulus conditions was compared regardless of the prior expectation conditions. Therefore, a direct comparison of the pursuit variance across prior expectations was not possible. In the present study, we tested whether different prior expectation conditions indeed contributed to the modulation of the pursuit variance in a single day’s experiment. For this purpose, we obtained at least 150 trials (up to 300 trials) for the central direction that is common among the narrow-prior and wide-prior blocks. After removing trials with saccades, poor pursuit initiation, and outliers (see section “Materials and Methods”), we included each day’s data for further analysis only if the central direction condition was valid in at least 70 trials so that robust estimation of the SD of the smooth pursuit directions may be achieved. We accumulated 60 days’ data from monkey A and 50 days’ data from monkey B. Figures 2D,E summarize the comparison of the SD in the wide-prior and the narrow-prior block. Most of the data points in both monkeys are below the unity line, implying that the SD in the narrow-prior block was smaller than that in the wide-prior block. This difference was significant only in the low-contrast case although the change trend was similar in the high-contrast case (mean SD for monkey A: 13.99° vs. 11.59° for the low-contrast case, *t*-test *p* = 4.8 × 10^–15^, 8.34° vs. 8.12° for the high-contrast case, *t*-test *p* = 0.06; mean SD for monkey B: 11.84° vs. 9.15° for the low-contrast case, *t*-test *p* = 6.7 × 10^–12^, 5.72° vs. 5.58° for the high-contrast case, *t*-test *p* = 0.09). Therefore, we concluded that the influence of the prior expectation about motion direction on the precision of smooth pursuit directions was consistent with the prediction of the Bayesian observer model.

### Correlation Between the Changes in Accuracy and Precision of Smooth Pursuit Direction by Prior Direction Expectation

**FIGURE 3:**
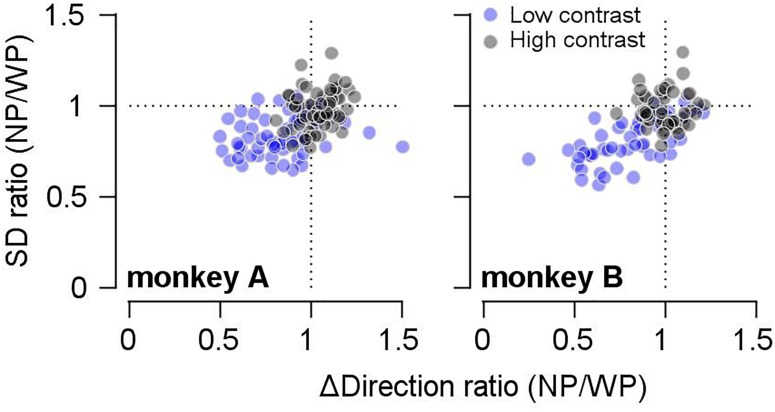
Relationship between changes in bias and SD by prior direction expectations. The *x*-axis shows the ratios of direction differences between the two prior conditions, and the *y*-axis shows the ratios of SD between the two prior conditions. Gray filled circles show the data when the high-contrast patch was a target stimulus, and blue filled circles show the data when the low contrast-patch was a target stimulus.

We observed that prior expectation increased both the bias (reduction in accuracy) and the reliability (increase in precision) of smooth pursuit direction, especially when the given motion information was imprecise. If this trade-off was due to the prior direction expectation, the size of the bias should be correlated with the precision of pursuit direction across different days’ experiments. Therefore, we tested whether this was the case. To quantify the effect of the prior expectation on the reliability of pursuit directions, we calculated the SD of the pursuit direction (for the common prior direction) in the narrow-prior and wide-prior cases. Then, we obtained their ratio (SD_narrow_/SD_wide_). Moreover, regarding the effect of the prior on the bias, we calculated the direction distance ratios (see above) in the narrow-prior and wide-prior cases, and we obtained their ratio. Then, we tested whether the two measures were correlated (Figure 3). In monkey A, the two measures were significantly correlated regardless of the stimulus/contrast pattern (Figure 3, left panel. High contrast spearman’s rho = 0.29, *p* = 0.024, low-contrast rho = 0.29, *p* = 0.023). In monkey B, the two measures were correlated in the low-contrast case (Figure 3, right panel. Spearman’s rho = 0.678, *p* = 1.9 × 10^–7^). They were not correlated when the contrast of the pursuit target was 100% (rho = -0.07, *p* = 0.617). These significant correlations suggest that the change in reliability and bias in the narrow-prior and wide-prior blocks may be due to a common mechanism.

### Bayesian Observer Model Explains the Effect of Prior Expectation on the Accuracy and Precision of Smooth Pursuit Eye Movements

We demonstrated that the modulation of the accuracy (bias) and precision (variability) of the initiation of smooth pursuit eye movements by prior directional expectation is consistent with the prediction of the Bayesian observer model. Subsequently, we tested whether the Bayesian observer model indeed explained the change in the precision and bias of smooth pursuit eye movements. For this computational analysis, we assumed that the prior probability and the likelihood function follow the Gaussian distribution. Therefore, the resultant posterior probability also follows the Gaussian distribution (see section “Materials and Methods”). We considered five pursuit directions, two prior conditions, and two stimulus conditions. For each stimulus condition, we calculated the mean and the SD of the pursuit direction using the previously mentioned decomposition method (see section “Materials and Methods” for details). Then, we compared them with the maximum *a posteriori* estimates from the Bayesian observer model [Equations (4) and (5) in section “Materials and Methods”]. In the model, the SD of the likelihood functions (five directions), the SD of prior probabilities (two parameters), and the contrast/stimulus factor (one parameter) were estimated (eight free parameters) from the 12 means and 12 SDs of the pursuit directions (three directions × two stimuli × two prior conditions).

**FIGURE 4:**
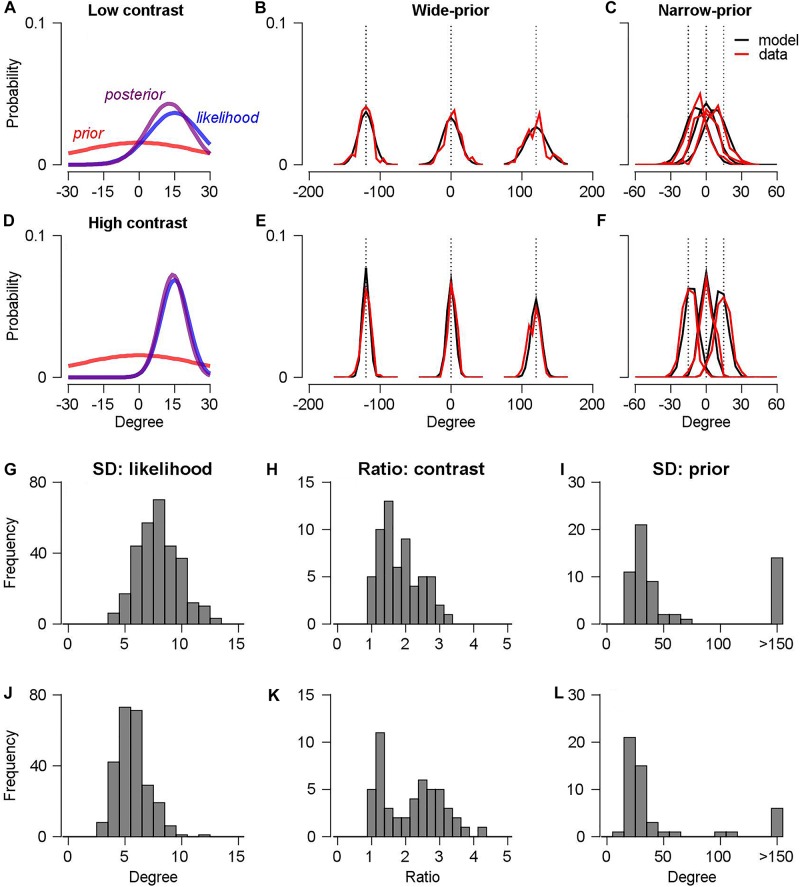
Bayesian observer model of smooth pursuit eye movements. **(A,D)** Estimated prior probability distribution (red color), likelihood function (blue line), and posterior probability distribution (purple line) in the low-contrast **(A)** and the high-contrast case **(D)**. **(B,E)** The predicted distributions of pursuit directions from the Bayesian observer model in the wide-prior case. Black lines show the model predictions, and red lines show the smooth pursuit data in the low-contrast **(B)** and high-contrast case **(E)**. **(C,F)** The predicted distributions of pursuit directions in the narrow-prior case. Figure formats are the same as in panels **B,E**. **(G,J)** Histograms of estimated parameters for the SD of the likelihood function for monkeys A(G) and B(J). **(H,K)** Histograms of estimated contrast ratio factors from the two monkeys. **(I,L)** Histograms of estimated parameters for the SD of the prior probability distribution for the two monkeys.

Figure 4A shows the estimated prior distribution, likelihood function, and posterior distribution of an example session in a narrow-prior block when the stimulus contrast was low. Figure 4D shows the same information when the stimulus contrast was high. In this example, the posterior distribution was shifted toward the prior direction in the low-contrast case, whereas the posterior distribution did not significantly change in the high-contrast case. This was the typical observation in the experiments. Figures 4B,C,E,F show the corresponding distributions of the pursuit directions (red traces) in the wide-prior block (Figure 4B: low contrast, Figure 4E: high contrast) and the narrow-prior block (Figure 4C: low contrast, Figure 4F: high contrast), with the predicted probability distributions from the Bayesian observer model (black traces). Overall, the model explained 98 ± 13% of the variance of the pursuit direction mean and 84 ± 15% of the variance of the pursuit direction SD for monkey A; the corresponding values for monkey B were and 99 ± 0.1% and 92 ± 7%. Therefore, the assumption of the Bayesian direction prior explains the changes in the mean and the SD of the pursuit direction. The estimated parameters themselves are consistent with the behavioral data that we observed from the smooth pursuit direction traces. The average SD of the likelihood function across the 60 sessions for monkey A was 7.94° (Figure 4G) and the contrast factor was 1.83 (Figure 4H). Therefore, the SD of the likelihood function in the low contrast/stimulus case was approximately 1.8 times as high as the SD of the likelihood function in the high-contrast case. The SD of the prior probability distribution was approximately 34.5° (median value approximately four times as high as the SD of the likelihood function in the high-contrast case, Figure 4I) in the narrow-prior block, whereas the estimated prior SD in the wide-prior block was particularly large; this corresponds to a flat-prior probability distribution (median *SD* = 10^6^°, data not shown). In monkey B, the overall change trends of the estimated parameters across the 50 sessions were similar to those for monkey A. The SD of the likelihood function was averaged to 5.63° (Figure 4J), which was significantly smaller than that in monkey A (two-sample *t*-test, *p* < 10^–40^). Given that monkey B’s smooth pursuit velocity in directions was more reliable than that of monkey A (the average SD for monkeys A and B was 8.34° and 5.72°, respectively, in the high-contrast, wide-prior case, Figures 2D,E), this result is expected. The average contrast factor was 2.17 (Figure 4K), which was slightly higher than the average value for monkey A. However, given that we used lower-contrast stimulus with/without directional noise in monkey B (8% vs. 8%, 10%, or 12%), the higher contrast factor is also expected. The SD of the prior probability distribution in the narrow-prior block was approximately 25.7° (median value approximately 4.5 times as high as the SD of the likelihood function in the high-contrast case, Figure 4L). In the wide-prior block, the SD of the prior probability distribution was also significantly large (median *SD* = 10^5^°, data not shown). These results demonstrated that the direction of the feedforward, open-loop smooth pursuit eye movement can be understood as a resultant motor behavior under Bayesian inference. The proper configuration of motion directions enabled the monkeys to develop a prior and to switch the prior expectation in a single day’s experiment.

### Tracking the Temporal Dynamics of Bayesian Inference From Smooth Pursuit Eye Movements

**FIGURE 5:**
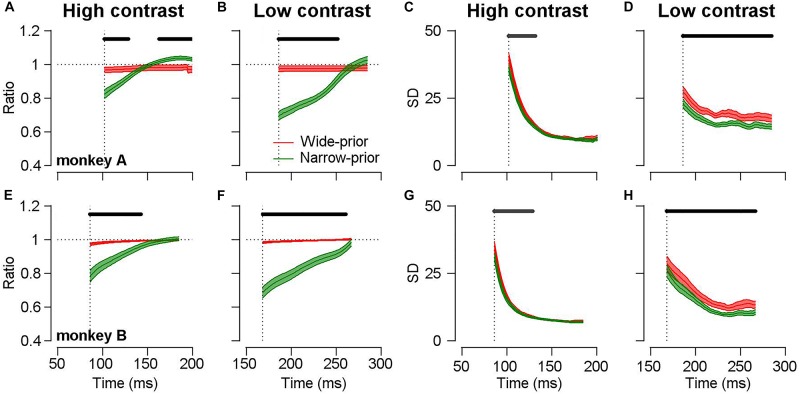
Time-dependent changes in bias and precision under different prior conditions. Black lines above each figure show the time-clusters where values are significantly different across the prior conditions (cluster-based permutation test, *p* < 0.05). Colored error bands show the standard error. Vertical dotted lines show the average pursuit latency of individual animals for each stimulus condition. **(A,E)** Time-dependent changes in direction difference ratios in the high-contrast case. Red color shows the data in the wide-prior case and green color shows the data in the narrow-prior case. **(B,F)** Time-dependent changes in direction difference ratios in the low-contrast case. **(C,G)** Time-dependent changes in the SD of pursuit directions in the high-contrast case. **(D,H)** Time-dependent changes in the SD in the low-contrast case.

The open-loop smooth pursuit eye movements are known to exhibit the characteristics of feedforward processes before any feedback information affects the behavior (Lisberger et al., 1987). Therefore, by carefully analyzing the initiation of smooth pursuit, we can observe the temporal dynamics of the underlying feedforward neural processes for sensory–motor behavior. As we could induce the monkeys to switch prior expectation about motion direction in a single day’s experiment, we were able to observe the interaction between sensory signal and prior expectation as a function of time. If changes in the pursuit direction bias and precision indeed depend on the strength of motion representation (the width of the likelihood function), then the pursuit direction bias and precision should be modulated not only by the luminance contrast/stimulus patterns but also by the time required for the pursuit system to process the sensory motion information. This prediction is consistent with the finding that even when motion information in a given stimulus is precise, insufficient time for processing this information accentuates the effect of prior expectation on the smooth pursuit (Figure 5). At the very early stage of pursuit initiation (less than 150 ms from motion onset), there are clear differences in behavioral bias (Figures 5A,E, attraction toward the prior direction) and behavioral precision (Figures 5C,G, SD of pursuit directions) across the prior conditions (two-sided cluster-based permutation test, *p* < 0.05) under high-contrast stimulus. The difference becomes insignificant and tends to vanish at approximately 150 ms from motion onset. In the low-contrast case, the differences in behavioral bias and precision were maintained until the end of the open-loop period (Figures 5B,D,F,H). The time-dependent changes in the pursuit direction bias and precision can be readily explained if the neural implementation of the likelihood function becomes precise, as the visual system has sufficient time to integrate the incoming motion information and form a solid neural representation. One may interpret this as a result of the change in the prior expectation rather than the likelihood function. For example, a previous study demonstrated that the assumption of dynamic bias signal by the prior probability in bounded evidence accumulation models was better in explaining behavior choices for motion discrimination tasks than the static bias signal assumption (Hanks et al., 2011). However, as the authors of that study stated, this dynamic bias signal could be due to the conversion from probability distributions to decision variables. Equation 3 considers Bayesian inference at one point in time. If we now consider the equivalent evidence accumulation over time (with noisy data), we end up with a form of Bayesian filtering. Crucially, for our arguments, the rate of evidence accumulation is proportional to the precision of the sensory evidence. Technically, under linear assumptions, this precision is known as the Kalman gain (Ghahramani and Roweis, 1999; Perrinet et al., 2014; Baltieri and Buckley, 2018). A straightforward prediction of these optional schemes would be an increased rate of evidence accumulation – and neuronal correlates – when the precision of sensory evidence was high (or the precision of prior beliefs was low). This would translate into a reduced latency of the sort that we observed.

### Effect of Single-Trial Adaptation

**FIGURE 6:**
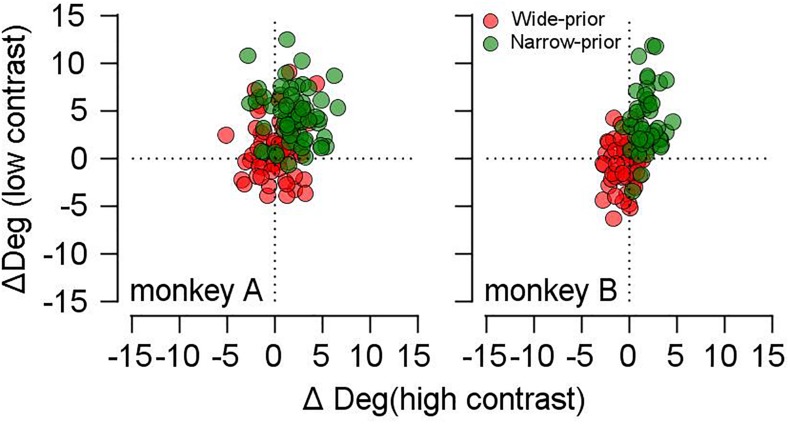
Effect of single-trial adaptation on the current trial pursuit direction. Red filled circles show the values in the wide-prior case, and green filled circles show the values in the narrow-prior case. The *x*-axis shows the data in the high-contrast case, and the y-axis shows the data in the low-contrast case.

A previous study raised the possibility that pursuit adjustment by prior expectation could originate from single-trial adaptation and eventually be the mechanism for the formation of the Bayesian prior (Darlington et al., 2017). In the current study, single-trial adaptation may be an issue because it can trivially explain the change in the SD of smooth pursuit across the prior conditions. For example, if eye velocity traces were attracted more toward the previous trial direction in the wide-prior case than in the narrow-prior case, then the precision increase in the latter could be easily explained. Therefore, we selected the pursuit data in the common central direction and divided the trials according to the directions of the prior trial. We considered only the trials whose previous trial directions were different from the current trial direction. We averaged trials whose previous trials were in counterclockwise direction from the current trials and averaged other trials whose previous trials were in clockwise direction from the current trials. Then, we calculated the difference between the two. If this value was positive, the current trial pursuit direction was attracted toward the previous trial motion direction; otherwise, the current trial pursuit direction was repelled from the previous trial motion direction. In both monkeys, these values were significantly higher than zero in the narrow-prior case regardless of stimulus contrasts (Figure 6, green circles, mean values = 2 for high contrast, 4.43 for low contrast in monkey A, *t*-test *p* < 10^–9^ for both stimulus conditions, 1.9 for high contrast, 3.9 for low contrast in monkey B, *t*-test *p* < 10^–10^ for both stimulus conditions). In the wide-prior case (Figure 6, red circles), the values were significantly higher than zero in the low-contrast case for monkey A (mean value = 1.18, *p* = 0.005) but significantly smaller than those in the high-contrast case (two-sample *t*-test, *p* = 2.4 × 10^–8^). In monkey B, the values were significantly smaller than zero in the high-contrast case (mean value = -0.55, *t*-test *p* = 0.002). Therefore, the attraction toward the previous trial was stronger in the narrow-prior case. If the change in the pursuit direction variability across the prior conditions was the result of single-trial adaptation, the effect should be stronger in the wide-prior block. However, we observed the opposite. In summary, there was evidence of single-trial adaptation, but this could not explain the SD reduction in the narrow-prior block.

## Discussion

In this study, we developed an experimental paradigm that enabled us to observe the effect of prior expectation about motion direction on the precision and accuracy of the initiation of smooth pursuit under identical sensory stimulus conditions. We observed directional bias in the pursuit velocity traces toward the prior direction and a significant reduction in trial-by-trial variability. We also reported the effect on the temporal dynamics of bias and variability. These typical properties of Bayesian inference were confirmed by the computational modeling of the Bayesian observer model.

### Cognitive Control and Bayesian Inference in Smooth Pursuit Eye Movements

The incorporation of bottom–up sensory signals with top–down cognitive signals for behavior control is an important issue in oculomotor behavior. The effect of the predictive, anticipatory aspects of smooth pursuit eye movements has been extensively investigated (Kowler et al., 2014). It has been demonstrated that an informative cue about the future direction of the target motion can induce anticipatory smooth pursuit eye movements (de Hemptinne et al., 2006, 2008). Other studies demonstrated that the visual motion that is seen or tracked in the recent past can cause anticipatory smooth pursuit (Kowler et al., 1984; Heinen et al., 2005; de Hemptinne et al., 2007; Collins and Barnes, 2009). Although the influence of experience-dependent prior knowledge on visually guided smooth pursuit eye movements has been known, the quantitative description, and theoretical ground for explaining the interaction between sensory and predictive signals was lacking. The Bayesian inference framework provided the theoretical ground for explaining this interplay.

The Bayesian observer model has recently attracted considerable attention and provided a comprehensive framework that explains the underlying mechanisms of perceptual inference (Ma, 2012). A number of studies sought to explain the information processes in smooth pursuit eye movements using the Bayesian inference framework. Yang et al. (2012) demonstrated that the interaction between the strength of the sensory stimulus and the prior knowledge about motion can be explained by the Bayesian observer model and provided a plausible neural implementation. In that study, however, several days were required for the monkeys to develop a direction prior, and therefore a comparison of the effect of the prior expectation on smooth pursuit eye movements in a controlled manner was not possible. In subsequent studies, Darlington et al. (2017) studied the fast development of priors for speed and direction. They reported that the two fast-adapting priors can be explained by direction-specific gain control in sensory-motor transmission through computational analysis. In humans, several studies investigated the weighted integration of retinal and extra-retinal signals in smooth pursuit. Orban de Xivry et al. (2013) demonstrated that Kalman filtering can reproduce the major properties of visually guided smooth pursuit and anticipatory smooth pursuit. A later study demonstrated that the reliability-based integration of prior expectation with visual information occurred not only in smooth pursuits but also in catch-up saccades (Deravet et al., 2018). Similarly, Bogadhi et al. (2013) demonstrated that the reliability-weighted dynamic integration of retinal and extra-retinal signals can explain pursuit behaviors during target blanking. In those studies, the effect of the prior expectation on average pursuit traces was successfully demonstrated, but the effect on the trial-by-trial variability of sensory-motor behavior, which is another important aspect of the Bayesian inference (Ma, 2012), was not systematically investigated. In the present study, we demonstrated the interaction between the strength of the motion stimulus and the prior not only in the directional biases but also in the reliability of the smooth pursuit.

### Origin of the Bayesian Prior in Smooth Pursuit Eye Movements

Several recent studies demonstrated the formation of prior knowledge by a recent history of stimulus presentation (Verstynen and Sabes, 2011; Darlington et al., 2017; Deravet et al., 2018). In (Darlington et al., 2017; Deravet et al., 2018), the effect of the previous trial on the speed of the current trial was emphasized in smooth pursuit, and this single-trial adaptation was proposed as the mechanism for the prior formation and the competition between sensory evidence and prior expectation. In the present study, the analysis of the effect of the direction of the previous trial on the direction of the current trial suggested that the prior knowledge that monkeys developed had at least two components. One component may originate from single-trial adaptation, which can be explained by simple directional gain modulation. The other component may originate from the accumulation of knowledge about the experimental conditions through longer-term training. In our data, the changes in the bias can be explained by simple gain modulation: the stronger effect of the prior expectation in the narrow-prior block can be explained by direction-specific gain modulation (see Figures 10B,C in Darlington et al., 2017). However, the change in the reliability of the smooth pursuit direction by prior expectation cannot be explained by single-trial adaptation. If single-trial adaptation was the underlying mechanism for the reliability changes, the SD of the pursuit directions should be smaller in the wide-prior than in the narrow-prior case because the single-trial adaptation was greater there; however, we observed the opposite (Figure 6). We do not know if the single-trial adaptation and the long-term direction prior that we observed here and in (Yang et al., 2012) share the same neural mechanism. The acquisition of the long-term prior could have been triggered by single-trial adaptation, but the mechanism and location of the final neural representation for the single-trial adaptation and long-term prior may be completely different.

The integration of prior expectation and sensory information in smooth pursuit eye movements was readily explained by the Bayesian observer model, but we still do not have sufficient knowledge about the neural mechanisms underlying this integration in smooth pursuit behavior. Several studies investigated the effect of prior expectation and the underlying neural substrates that are involved in pursuit-based extra-retinal information. Some recent studies demonstrated that neural activity in the supplementary eye fields encoded the expected future direction of target motion (de Hemptinne et al., 2008) and guided the anticipatory smooth pursuit eye movements (Missal and Heinen, 2004). Neural activity in the frontal eye field smooth eye movement region (FEF_SEM_) is involved in controlling smooth pursuit eye movements [e.g., anticipatory (MacAvoy et al., 1991) and predictive pursuit (Fukushima et al., 2002), and gain modulation (Tanaka and Lisberger, 2001, 2002a)]. The current understanding of smooth pursuit eye movements emphasizes that the neural activity in FEF_SEM_ modulates the gain of visual-motor transmission (Tanaka and Lisberger, 2001, 2002b; Nuding et al., 2009; Lisberger, 2015). Therefore, the gain modulations implemented in FEF_SEM_ have been implicated as the loci of realization of the Bayesian prior (Yang et al., 2012; Darlington et al., 2017, 2018). Accordingly, the optimal integration of sensory evidence and prior knowledge has corresponding neural areas and circuit mechanisms in the smooth pursuit eye movement system: sensory motion information represented in the middle temporal area (area MT) is multiplicatively modulated by the activity of FEF_SEM_, and this gain-modulated motion information is transmitted to the downstream motor areas. In this approach, neural activity in area MT may represent only the likelihood function, and FEF_SEM_ may represent the prior information and the posterior probability distribution. A recent study supported this view. Darlington et al. (2018) demonstrated that the spontaneous ramping activity in FEF_SEM_ indeed represented the prior expectation of speed and the evoked responses represented the reliability-weighted integration of the prior and the likelihood function.

Although simple gain modulation was successful in explaining the bias of the smooth pursuit eye movements by prior expectation, it cannot fully explain the changes in trial-by-trial variability. In addition to the gain modulation, if neural activity in FEF_SEM_ represents the posterior distribution, the reduction in pursuit variability should be the result of decoding population neural activity of FEF_SEM_. Therefore, it is possible that there is a population-level noise reduction in FEF_SEM_ neural responses, for example, the reduction in trial-by-trial correlation between neurons (Zohary et al., 1994; Cohen and Maunsell, 2009; Mitchell et al., 2009) or the variability of spiking (Fano factor) (Mitchell et al., 2007). Given that human neuroimaging studies reported the enhancement of neural representation in sensory cortical areas by prior expectation (Kok et al., 2012, 2013), it is also possible that the neural representation of motion direction in area MT may be likewise improved. Then, the reduction in the pursuit direction variance could have neural origin in area MT population neural activity, e.g., changes in the direction tuning, neuron–neuron correlations, and Fano factor. Any of these changes can be the neural components that contribute to the modulation of pursuit direction variation and the generation of direction bias by prior expectation.

In this work, we have focused on the Bayesian brain hypothesis and ideal Bayesian observer assumptions. However, we have been examining behavioral responses in terms of eye movements. This means that we have had to make some strong assumptions about eye movements in terms of reporting posterior beliefs following Bayesian synthesis. This is an interesting area – usually addressed under the rubric of active inference. Here, we have assumed that the overt behavior directly reporting the MAP estimate and standard deviation. Other accounts would consider the posterior precision (which behaves in a similar way). Furthermore, one can imagine sampling behavioral responses from posterior beliefs, which would provide an alternative model of behavior. We will consider the alternative formulations of action selection under Bayesian assumptions in future work; however, we anticipate the basic conclusions of the current analysis – about evidence accumulation and perceptual synthesis – will be exactly the same.

## Data Availability Statement

The datasets generated for this study are available on request to the corresponding author.

## Ethics Statement

The animal study was reviewed and approved by the Sungkyunkwan University Institutional Animal Care and Use Committee.

## Author Contributions

SK and JL: conceptualization, methodology, and writing – original draft. SK and JP: investigation. SK, JP, and JL: formal analysis, and writing – review and editing. JL: funding acquisition.

## Conflict of Interest

The authors declare that the research was conducted in the absence of any commercial or financial relationships that could be construed as a potential conflict of interest.

## Footnotes

1 https://sites.google.com/a/srscicomp.com/maestro/
